# Impact of age on the postural stability measured by a virtual reality tracker-based posturography and a pressure platform system

**DOI:** 10.1186/s12877-022-03195-0

**Published:** 2022-06-18

**Authors:** Huey-Wen Liang, Shao-Yu Chi, Tzu-Ling Tai, Yue-Hua Li, Yaw-Huei Hwang

**Affiliations:** 1 Department of Physical Medicine and Rehabilitation, National Taiwan University Hospital and College of Medicine, Taipei, Taiwan, Republic of China; 2 Department of Physical Medicine and Rehabilitation, National Taiwan University Hospital, No 7, Chong-Shan South Road, Taipei, 100 Taiwan, Republic of China; 3 Department of Physical Medicine and Rehabilitation, National Taiwan University Hospital Bei-Hu Branch, Taipei, Taiwan, Republic of China; 4 Institute of Environmental and Occupational Health Sciences, College of Public Health, National Taiwan University, Taipei, Taiwan, Republic of China; 5 Master of Public Health Program, College of Public Health, National Taiwan University, Taipei, Taiwan, Republic of China

**Keywords:** Computerized posturography, Elderly, Outcome measure, Postural balance

## Abstract

**Background:**

Center of pressure (CoP) parameters are commonly used to evaluate age-related changes in postural control during standing. However, they mainly reflect ankle strategies and provide limited information about hip strategies, which are essential for postural control among the aged population. Body displacement at the lumbar level (LD) can be used as a proxy for hip strategies.

**Objectives:**

We set up a virtual reality tracker-based posturography to measure LD and compared the CoP and LD parameters in two age groups to explore the roles of ankle and hip strategies during bipedal stance.

**Methods:**

Twenty-seven older healthy participants (63.8 ± 7.1 years old) and 27 younger controls (31.7 ± 9.9 years old) performed four standing tasks with their postural steadiness measured simultaneously with both systems under four stance conditions (combination of eyes-open/eyes-closed and wide-based/narrow-based). Five parameters were calculated from the trajectories of the CoP and LD. The difference in the parameters between two groups was analyzed with the Mann–Whitney U test. The discriminative ability of the parameters from the two systems was computed by the receiver operating characteristic curve analysis and area under the curve (AUC). We also used the intraclass correlation coefficient (ICC) to assess the correlation between two measures.

**Results:**

Most of the parameters obtained from both systems were significantly different between the younger and older groups. Mean velocity in the medial–lateral and anterior–posterior directions could effectively discriminate age-related changes, especially with the LD parameters. The receiver’s operation curve analysis gained the largest AUC (0.85 with both systems) with mean velocity in the medial–lateral direction during narrow-based standing with eyes closed. Meanwhile, we observed a low correlation between parameters obtained from the two methods in velocity measures, with the lowest ICC in the mean velocity in the medial–lateral direction in the older group (ICC = 0.34 ~ 0.41).

**Conclusion:**

Both systems could differentiate age-related changes in postural steadiness, but with dissociated information about mean velocity, especially the mean velocity in the medial–lateral direction in the older group. The results support the complimentary role of using tracker-based posturography to understand the effect of age on the mechanisms of postural control.

## Background

Balance control is a prerequisite for the execution of daily activities, and encompasses the acts of maintaining, achieving or restoring the line of gravity within the base of support [1]. It is a critical part of clinical assessments or screening in rehabilitation. The ability of an individual to maintain postural control decreases with aging and the decline in postural control ability is associated with an increased incidence of falls [2, 3], which is one of the major causes of morbidity, disability and mortality in elderly people. Postural stability may decrease as early as in the fifth decade of life and declines rapidly after 70 years of age, depending on the tasks used for testing [4, 5]. The evaluation of balance ability is therefore an essential component for the risk assessment and intervention to prevent falls among the elderly [6].

Age-related changes in postural control ability can be documented in several instrumental and noninstrumental approaches, and instrumental methods are advantageous for providing quantitative data regarding control mechanisms exerted by older and younger populations [7]. The ankle strategy is generally used in situations of unperturbed posture and slow or low amplitude perturbations. Meanwhile, the hip strategy is employed in circumstances of fast or large amplitude perturbations [8, 9]. The gradual decline in ankle stabilizer muscle strength in older people results in reliance of the hip strategy with late activation of trunk and thigh muscles, especially when a stable upright posture is threatened. Centers of pressure (CoP), representing the projection on the ground plane of the centroid of the vertical force distribution and usually recorded by a kinetic device, is modulated by the ankle strategy.

The parameters computed from the CoP trajectories characterize multiple aspects of postural steadiness in the time (distance, area, and hybrid) and frequency domains [10]. However, the relative sensitivity of these measures to detect age-related changes in postural steadiness may vary considerably. The most commonly used measures are time-distance domain parameters (mean distance, root mean square distance and mean velocity) and time-domain area measures (the 95% confidence ellipse area). One meta-analysis showed that elderly people (≥ 65 y/o) have higher range (the distance between the most distant points of CoP displacement) and velocity (the total length divided by the time duration) than young people (18–35 y/o) in both the medial–lateral (ML) and anterior–posterior (AP) directions, with also higher degree of variability, implying different balance control capacities [11]. The difference of CoP displacements between elderly and young people during standing is reported to range from 30–40% in the eyes-open (EO) condition and 20% to 50% in the eyes-closed (EC) condition. For velocity, the age difference ranges from 30–50% in the EO condition and approximately 50% in the EC condition. The impact is more evident on velocity than on range and more evident in the ML direction than in the AP direction. In addition, deprivation of vision, changing standing surface or reducing stance width could amplify the impacts of aging on postural stability [10, 12].

Nonetheless, increases in CoP parameters, such as the displacement length, area, displacement, and velocity, are not necessarily associated with postural instability, but possibly reflect a stable control system in which the CoP makes frequent postural corrections to stabilize the center of mass (CoM), as long as the CoP does not approach the limits of the base of support [13, 14]. Moreover, the older individuals tend to use more of a hip strategy than younger individuals [15], which may not be able to be fully characterized by CoP measurements [14]. The control strategies are affected by not just age but also several factors, such as sex, the base of stance and visual condition [12, 14, 16]. A simultaneous measure of the CoP, center of gravity (CoG) and electromyography suggests that the ankle joint alone did not provide sole control of standing balance [14]. Older adults have greater postural sway with mixed hip-ankle activation in a narrow base of support without external perturbations, while younger adults accommodate for the increased postural requirements only by increasing the activity of ankle muscles [16]. However, muscle activity of hip agonists is not consistently observed in older adults [17], resulting in suboptimal effectiveness of hip strategy to compensate for the decline of ankle strategy. To characterize postural strategies exerted by different body segments beyond an ankle strategy, several approaches are available, including estimation of CoM acceleration from a force platform system recording [18] and using a motion capture system [19], electromyography [19], laser displacement sensors [20], computerized posturography [21], and electromagnetic sensors [22]. Meanwhile, we set up an easy and inexpensive method to measure body displacement at lumbar level (LD) by using a lumbar tracker and a commercial virtual reality (VR) system (VIVE Pro, by HTC, Inc. Taiwan). The concept of the VIVE tracker-based system is similar to that of a swaymeter [23], and can track postural sway trajectories in static or dynamic conditions. A recent study also shows that the measurements obtained by a tracker placed on the pelvis are reasonably representative of laboratory-based measurements of CoM displacement [24]. The test–retest reliability of the LD parameters obtained by the VIVE tracker-based system was moderate to high (intraclass correlation coefficients, ICCs = 0.56 ~ 0.90) [25]. In addition, the measurements against the CoP parameters were mostly, but not consistently, highly correlated across the four different standing conditions with EO/EC and feet together/apart among a group of young participants (Pearson’s correlation coefficient = 0.42 ~ 0.96). The study observed a tendency for a lower correlation in velocity-ML than other measures, and a relatively low correlation implied different information about postural control provided by these two systems. CoP-based measures are supposed to characterize ankle strategy, and tracker-based posturography obtained at the lumbar level can be a proxy for hip strategy. Increased velocity represents increased control activities, and CoP-based velocity-ML is sensitive to age-related change. Moreover, increased body sway velocity measured by a gravicorder is associated with fracture in community-dwelling elderly women [2]. Meanwhile, the sensitivity of tracker-based LD-based parameters to detect age-related change is to be explored.

In the current study, we measured postural stability in different stance conditions with both VR tracker-based posturography and a pressure platform system in younger and older participants to examine the impact of age on the postural stability measurements obtained by these two methods, which represented different trunk control strategies. We hypothesized that older adults have reduced postural stability, especially during challenging tasks, and this age-related change could be detected by both methods, with velocity parameters in the ML direction being the most discriminative for the age effect [26, 27]. We also hypothesized a lower correlation of ML velocity obtained by two measurements in the elderly since these methods were supposed to represent two postural control mechanisms and the elderly tend to rely on hip strategies to control stability in the frontal plane, as evidenced by the ML velocity. The results can help establish the clinical applicability of VIVE tracker-based posturography and be used in conjunction with CoP-based measures for fall risk assessments and interventions in elderly adults.

## Methods

### Study design and participants

This was a cross-sectional study. The participants were recruited by a convenience sampling from the university campus and the university hospital. A power analysis conducted using G*Power 3.1.9.7 was used to determine the required sample size to compare the two groups. Alpha was set to a value of 0.05, while the power was set to 0.90. A total sample size of 52 (26 per group) was required according to the mean and standard deviation of velocity-ML obtained from a previous study (11.4 ± 4.7 mm/s for the younger group and 18.0 ± 9.9 mm/s for the older group, effect size: 0.85) [28]. Fifty-four healthy adults aged between 20 and 82 years volunteered to participate and were divided into the older (55 years and older) and younger groups (less than 55 years old) (Table 1). Fifty-five years old was the upper limit in some studies for young and middle-aged groups [27, 29], and the decline of bipedal standing balance started from the fifth to sixth decades, depending on the task difficulties [4]. None of the participants had any known history of cognitive, cardiovascular, neurological or balance problems that would exclude them from the study. The two groups were similar in sex distribution, body height and body weight, but the older group had a higher body mass index (BMI) than the younger group. This study followed the guidelines of the Declaration of Helsinki and was approved by the Ethical Committee of the National Taiwan University Hospital (approval number: 201904129RINC, date of approval: 20/06/2019), and written informed consent was obtained prior to participation.

**Table 1:** Demographic data of all participants

Variables	younger group (*n* = 27)	older group (*n* = 27)	*p value
Male (n, %)	9 (33.3%)	11 (40.7%)	0.573
Age (years)			< 0.001
mean (standard deviation)	31.7 (9.9)	63.8 (7.1)	
Minimum–maximum	22 ~ 53	55 ~ 82	
Body height (cm)			0.635
mean (standard deviation)	164.4 (7.1)	163.4 (7.7)	
Minimum–maximum	153–180	149–177	
Body weight (kg)			0.123
mean (standard deviation)	58.0 (10.4)	62.8 (11.8)	
Minimum–maximum	44 ~ 88	45 ~ 93	
Body mass index (kg/m^2^)			0.033
mean (standard deviation)	21.4 (2.8)	23.5 (4.2)	
Minimum–maximum	17.0 ~ 30.8	17.4 ~ 36.3	

^*^p value: χ.^2^ test for categorical data and *t* test for continuous data

### CoP measurements

CoP was measured with the FDM-S pressure plate (Zebris Medical GmbH, Germany), with a sampling rate of 100 Hz. This system included 2,560 sensors over an area of 54 by 34 cm, yielding a resolution of approximately 8.7 sensors per square inch and an accuracy within 5%. The platform was connected to WinFDM software to compute the CoP positions during stance. We exported the CoP position data in the ML and AP directions as CSV files to calculate the CoP parameters.

### VIVE tracker-based posturography

LD was determined primarily by the VIVE Pro system (HTC, Inc. Taiwan), which included two infrared laser emitter units (lighthouses, SteamVR Base Stations V2.0) and three wireless trackers (Steam VR Tracking V1.0). One tracker was positioned on the posterior lumbar region at the pelvic level with a reference body frame, in which coordinates were established through the aid of two trackers on each dorsal foot. The setup was validated and described in detail in our previous research [25]. A custom C# script and the SteamVR (Valve Corp, Washington, USA) plugin for Unity3D were used to provide integration with virtual reality (VR) system to record the position and orientation of the trackers with a sampling rate of 100 Hz. The time series of displacements of the lumbar tracker in the AP and ML directions were used for further analysis.

### Procedure

Postural stability was assessed in a bipedal stance for two minutes under four conditions: wide-stance with eyes open (W-EO), wide-stance with eyes closed (W-EC), narrow-stance with eyes open (N-EO), and narrow-stance with eyes closed (N-EC). For the wide-stance stance, the participants stood with their heels 15 cm apart and their toes rotated outward by 25 degrees. For the narrow-stance, they stood with their feet side-by-side. The testing order of the four conditions was randomized and a repeated trial for each condition was conducted in the same order, yielding a total of eight 2-min tests for each participant. The participants were offered a seat and rested for at least one minute between tests. Simultaneous recordings from the VIVE tracker-based system and the pressure platform were obtained during each test. For both the wide-stance and narrow-stance, the participants stood at a fixed position on the pressure platform to standardize the posture and align the coordinate system. They were required to put their arms down at their sides and remain as stable as possible during the standing tasks. In the EO stance, the participants were asked to look at a fixed target on the wall 2 m ahead.

### Calculation of postural sway and CoP parameters

The time series position data from the platform system were used to calculate CoP parameters and those from the lumbar tracker were used as a proxy of trunk sway near the level of CoM to calculate the LD parameters. Both sets of data (CoP and LD) were passed through a fourth-order zero-phase Butterworth low-pass digital filter with a 5-Hz cutoff frequency in MATLAB (MathWorks Inc, Massachusetts, USA). The bivariate time series for the filtered CoP and LD data was defined by the AP and ML coordinates with respect to the origin of the coordinate system and used to compute the following parameters [10]:

- 1. The mean distances in the ML direction (distance-ML) and AP direction (distance-AP), which were derived from the AP and ML time series and represented the average distance of the CoP and LD relative to the origin of the coordinate system;
- 2. The mean velocity in the ML direction (velocity-ML) and AP direction (velocity-AP), which were computed with time and total length of the CoP or LD path (approximated by the sum of the distances between consecutive points) as the mean velocities of the CoP and LD in the ML and AP directions;
- 3. The 95% confidence ellipse area (AREA-CE) was the area of the 95% bivariate confidence ellipse for CoP and LD.

These parameters were chosen to represent the effectiveness of, or the stability achieved by, the postural control, and they belonged to five distinctive groups according to a correlation analysis for the CoP parameters (Pearson’s correlation coefficient > 0.9) [10].

### Statistical analysis

Five parameters obtained from the two two-minute tests were averaged for further analysis and the normality of the data was assessed using the Shapiro–Wilk test. The CoP and LD parameters were compared between the older and younger groups with the independent *t* test or Mann–Whitney U test if normality assumption was not met. The discriminative ability of these two measures was analyzed with receiver operating characteristic (ROC) analysis to compute the area under the curve (AUC), sensitivity and specificity. The optimal cutoff point was obtained with the higher AUC that best distinguished between the two age groups according to Youden’s index [30]. The AUC was interpreted as follows: 0.9 ~ 1.0 = excellent; 0.8 ~ 0.9 = good; 0.7 ~ 0.8 = fair; 0.6 ~ 0.7 = poor; 0.50 ~ 0.6 = fail [31]. We also computed the ICCs between the CoP and LD parameters for each stance condition and age group to evaluate not only the degree of correlation but also the agreement between these two measurements [32]. The computation was based on a two-way mixed-effects model for consistency, with an ICC higher than 0.90 as excellent, between 0.75 and 0.9 as good, between 0.5 and 0.75 as moderate, and less than 0.5 as poor [32].

Statistical analyses were performed using SPSS 15.0 for Windows (SPSS Inc, Chicago, USA) with p < 0.05 indicating statistical significance.

## Results

**Fig. 1:**
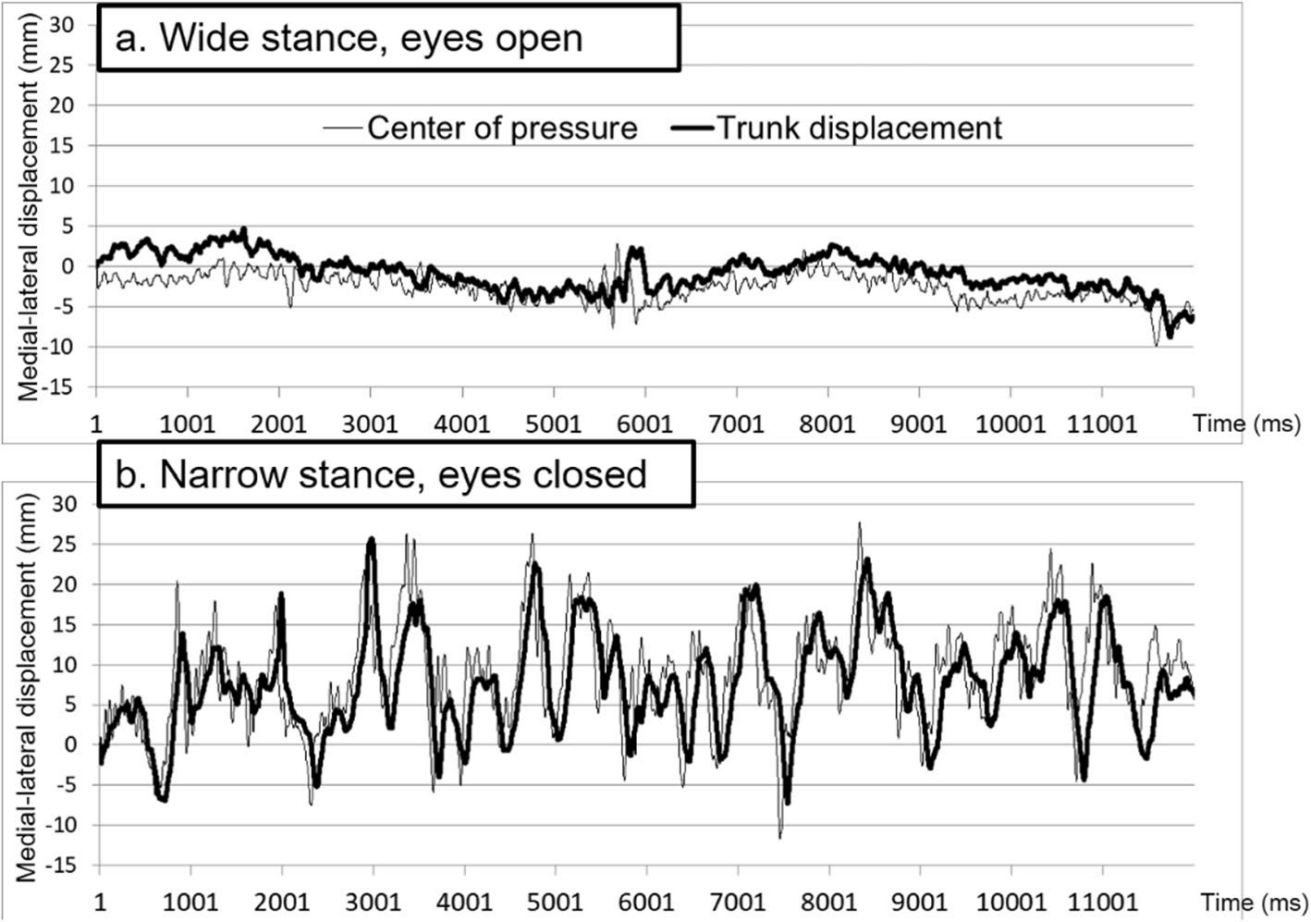
An example of the trajectories in the medial–lateral direction from the pressure platform and the VIVE-tracker system in a 74-year-old female participant

**Fig. 2:**
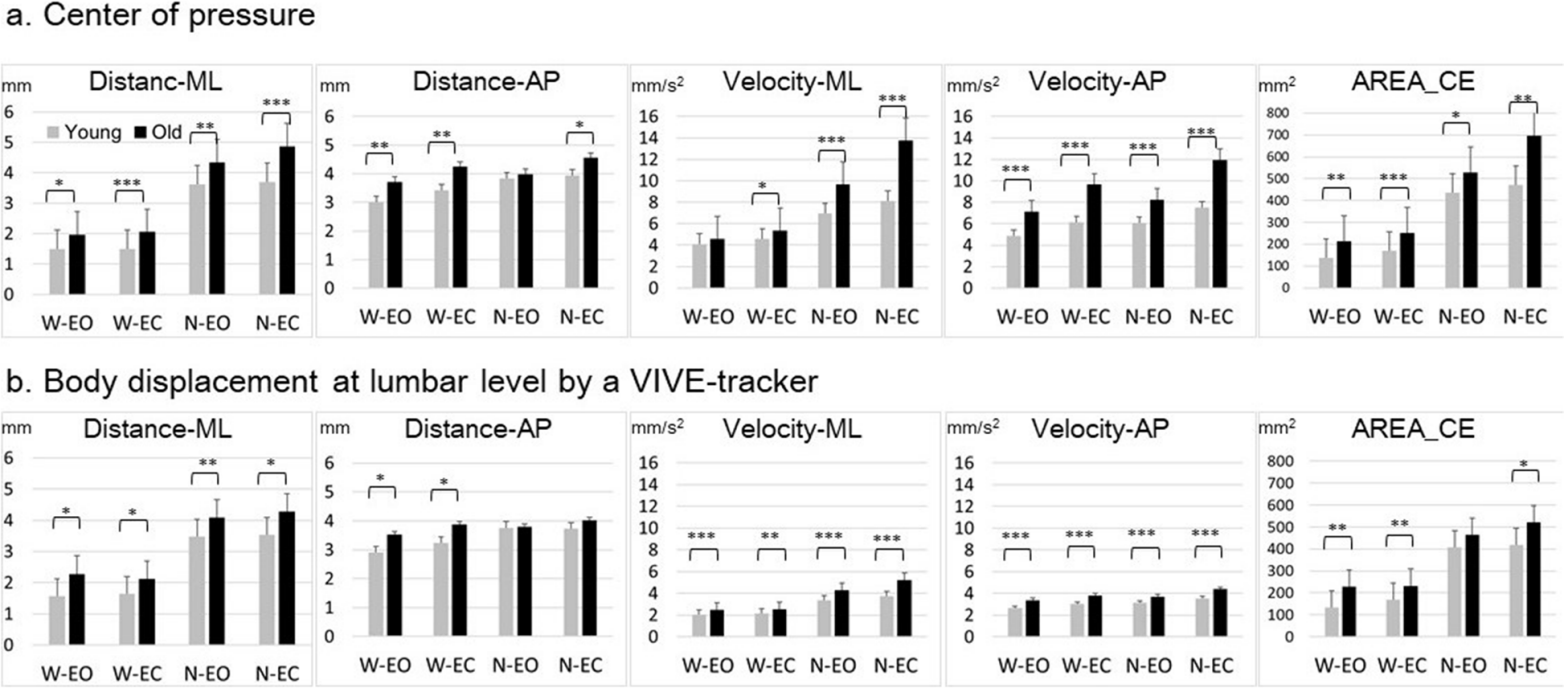
The median value and interquartile range of parameters from the pressure platform and the VIVE tracker-based system were compared between the younger and older groups. Note: Comparison between the younger and older groups with the Mann–Whitney test; * *p* < 0.05, **p≦0.01, ***p≦0.001. ML = medial–lateral direction; AP = anterior–posterior direction**;** AREA-CE: 95% confidence ellipse area

Since most of the CoP and LD parameters violated the assumption of normal distribution, the descriptive data were presented with median and interquartile range and analyzed with nonparametric methods if applicable. Figure 1 shows an example of the CoP and LD trajectories in the ML direction under two stance conditions for a 74-year-old female participant. There was a trend of increasing sway across the conditions in the following order: W-EO, W-EC, N-EO and N-EC (Fig. 2), and a larger amplitude of change from wide-stance to narrow-stance for parameters in the ML direction than the AP direction.

Regarding the effect of age, the older group had significantly larger CoP and LD parameter values than did the younger group with only a few exceptions. For the CoP parameters, the exceptions were the velocity-ML in the W-EO condition and distance-AP in the N-EO condition. For the LD parameters, the exceptions were the distance-AP in both the N-EO and N-EC conditions, and AREA-CE in the N-EO condition.

The discriminative ability of different parameters in four standing conditions was also evaluated with ROC analysis (Table 2). Less than fair discriminative ability to differentiate younger and older groups was observed with most distance measures from both systems. In contrast, most of the velocity parameters had fair to good discriminative ability, with the exception of velocity-ML of CoP during W-EO and W-EC conditions. For the LD parameters, velocity-ML and distance-AP had the largest and smallest AUCs respectively across all the stance conditions. Meanwhile, most of the LD parameters had higher sensitivity than specificity, with sensitivity as 0.52 to 0.96 and specificity as 0.48 to 0.85. This trend was less apparent for the CoP parameters.

**Table 2:** The results of receiver operating characteristic analysis of the center of pressure and trunk displacement parameters in differentiating the two age groups

		area under curve	*p* value	cut point	sensitivity	specificity
Center of pressure						
wide-based, eyes open	distance-ML	0.69	0.01	1.36	0.89	0.48
	distance-AP	0.70	0.01	4.43	0.59	0.78
	velocity-ML	0.64	0.08	4.51	0.56	0.81
	velocity-AP	0.81	0.00	5.8	0.70	0.93
	AREA-CE	0.71	0.01	103.85	0.89	0.55
wide-based, eyes closed	distance-ML	0.76	0.00	1.66	0.70	0.74
	distance-AP	0.72	0.01	3.82	0.70	0.78
	velocity-ML	0.68	0.03	5.12	0.59	0.78
	velocity-AP	0.81	0.00	6.81	0.81	0.71
	AREA-CE	0.78	0.00	158.25	0.81	0.71
narrow-based, eyes open	distance-ML	0.72	0.01	3.68	0.74	0.63
	distance-AP	0.55	0.53	3.89	0.52	0.67
	velocity-ML	0.82	0.00	7.94	0.78	0.78
	velocity-AP	0.78	0.00	7.13	0.67	0.81
	AREA-CE	0.66	0.04	382.03	0.78	0.59
narrow-based, eyes closed	distance-ML	0.75	0.00	4.71	0.63	0.85
	distance-AP	0.66	0.05	3.69	0.85	0.63
	velocity-ML	0.85	0.00	10.90	0.67	0.92
	velocity-AP	0.81	0.00	10.74	0.56	0.96
	AREA-CE	0.72	0.01	494.30	0.74	0.74
tracker-based trunk displacement						
wide-based, eyes open	distance-ML	0.69	0.02	1.36	0.85	0.48
	distance-AP	0.68	0.02	2.46	0.85	0.48
	velocity-ML	0.78	0.00	2.27	0.63	0.85
	velocity-AP	0.77	0.00	2.71	0.81	0.67
	AREA-CE	0.72	0.01	103.13	0.85	0.59
wide-based, eyes closed	distance-ML	0.70	0.01	1.67	0.81	0.60
	distance-AP	0.67	0.03	3.08	0.74	0.59
	velocity-ML	0.72	0.00	2.04	0.89	0.52
	velocity-AP	0.79	0.00	2.89	0.93	0.66
	AREA-CE	0.72	0.00	165.1	0.74	0.70
narrow-based, eyes open	distance-ML	0.71	0.01	3.04	0.89	0.52
	distance-AP	0.53	0.74	3.15	0.70	0.49
	velocity-ML	0.83	0.00	3.33	0.96	0.71
	velocity-AP	0.76	0.00	3.23	0.85	0.74
	AREA-CE	0.63	0.11	232.15	0.93	0.37
narrow-based, eyes closed	distance-ML	0.69	0.02	3.27	0.81	0.52
	distance-AP	0.62	0.14	3.35	0.78	0.59
	velocity-ML	0.85	0.00	4.38	0.78	0.85
	velocity-AP	0.77	0.00	3.89	0.74	0.82
	AREA-CE	0.67	0.04	391.2	0.67	0.70

*Note*: *ML* Medial–lateral direction, *AP* Anterior–posterior direction, *AREA-CE*: 95% confidence ellipse area

The ICC between the CoP and LD parameters showed good to excellent reliability of distance-AP_,_ distance-ML and area for both age groups (Table 3). Nevertheless, the velocity measures consistently had the lowest ICCs, especially in the older group (ICC = 0.34 to 0.51).

**Table 3:** The intraclass correlation coefficient between the center of pressure and tracker-based body displacement parameters

	Wide stance		Narrow stance	
Variable	Eyes open	Eyes closed	Eyes open	Eyes closed
Younger group				
distance-ML	0.72 (0.47 ~ 0.86)***	0.84 (0.67 ~ 0.92)***	0.84 (0.68 ~ 0.92)***	0.93 (0.84 ~ 0.97)***
distance-AP	0.94 (0.87 ~ 0.97)***	0.94 (0.86 ~ 0.97)***	0.89 (0.78 ~ 0.95)***	0.95 (0.89 ~ 0.98)***
velocity-ML	0.35 (-0.02 ~ 0.64)*	0.59 (0.28 ~ 0.79)***	0.43 (0.07 ~ 0.69)*	0.50 (0.16 ~ 0.74)**
velocity-AP	0.55 (0.22 ~ 0.76)**	0.64 (0.35 ~ 0.82)***	0.56 (0.23 ~ 0.77)**	0.49 (0.15 ~ 0.73)**
AREA-CE	0.76 (0.54 ~ 0.88)***	0.94 (0.87 ~ 0.97)***	0.94 (0.87 ~ 0.97)***	0.94 (0.88 ~ 0.97)***
Older group				
distance-ML	0.74 (0.51 ~ 0.87)***	0.77 (0.55 ~ 0.89)***	0.83 (0.65 ~ 0.92)***	0.89 (0.77 ~ 0.95)***
distance-AP	0.93 (0.84 ~ 0.97)***	0.89 (0.77 ~ 0.95)***	0.92 (0.84 ~ 0.96)***	0.90 (0.80 ~ 0.96)***
velocity-ML	0.34 (-0.04 ~ 0.64)*	0.36 (-0.02 ~ 0.65)*	0.41 (0.05 ~ 0.68)*	0.40 (0.03 ~ 0.67)*
velocity-AP	0.51 (0.17 ~ 0.74)	0.43 (0.06 ~ 0.69)*	0.41 (0.04 ~ 0.68)*	0.36 (-0.02 ~ 0.65)*
AREA-CE	0.89 (0.77 ~ 0.95)***	0.88 (0.76 ~ 0.94)***	0.93 (0.86 ~ 0.97)***	0.91 (0.81 ~ 0.96)***

*Note*: *ML* Medial–lateral direction, *AP* Anterior–posterior direction, *AREA-CE*: 95% confidence ellipse area; *p <0.05, **p ≦0.01, ***p ≦0.001

## Discussion

We evaluated postural steadiness and explored the age-related effects on postural stability in four stance conditions with two methods: a pressure platform and a VIVE tracker-based posturography, with the goals of testing their respective ability to differentiate younger and older age groups. The former method obtains CoP, which represents the vertical projection of the CoG on the transverse plane and is commonly used to document age-related changes [10, 33]. Meanwhile, the latter system includes a commercially available VR system with a tracker at the lumbar area to track the LD at a level near the CoM. The results showed that several CoP and LD parameters can differentiate postural steadiness between the older and younger groups. Velocity-ML recorded by tracker-based posturography had high sensitivity. In addition, the correlation and agreement between the CoP and LD parameters were high in the distance and area parameters, but low in the velocity parameters, especially in the older group and in narrow-based standing. These findings imply possible dissociation of hip and ankle strategies during a challenging standing task between younger and older groups and our system was able to provide additional information on CoP parameters.

The negative impact of aging on postural control has been the focus of many studies, because the impaired balance increases the risk of falls and leads to morbidities and mortality in this population [34]. Our study results agree with previous study findings, showing significantly less stability (larger distance-related measures and area) and a higher level of balancing-related “activity” (larger velocity measures) in elderly people than in young people [11, 35, 36]. The age difference between the two groups was around 30 years, which was lower than most of the other studies [11]. This finding again supports the discriminative validity of the tracker-based posturography. It also conforms the fact that declines in postural stability can be evident at ages as early as 50 years old, depending on the task difficulties [4, 37].

Among these five parameters from both systems, the differences between the age groups were more apparent in the velocity measures than in the distance and area measures and in the sagittal plane than in the frontal plane, which was consistent with the findings of several previous studies [10, 35, 36]. This finding can be explained by the fact that mean distance is related to the effectiveness of, or the stability achieved by, the postural control system, while mean velocity is related to the amount of regulatory activity associated with this level of stability [20]. This group of healthy subjects should not have great difficulty maintaining standing balance, but the elderly may require higher postural control activities to achieve similar level of steadiness to the young controls.

Most of the above age-related differences could be characterized by the parameters obtained from both systems, but the low ICC between the CoP and LD velocity parameters pointed to some difference between the two measurements. The ICCs for distance and area parameters from the two measurements were mostly good to excellent (ICC > 0.75) in both age groups across four standing conditions. Comparatively, the ICCs of the CoP and LD velocity parameters were mostly poor (< 0.5), especially in the older group. We suspected it to be related to the discrepancy of hip and ankle strategies in postural control characterized by two systems. From a clinical perspective, poor control of lateral stability is a major problem associated with increased risk of falling in elderly people [7], and age-related changes in hip abductor and adductor torques are important contributing factors [38]. Since ML displacement is mainly related to hip strategy [39], the velocity-ML of LD should be able to characterize the age-related change better than CoP parameters with narrow-based standing. This hypothesis was supported by the high discriminative ability of LD velocity-ML in N-EO and N-EC conditions according to the Mann–Whitney U test and ROC analysis. Velocity-ML of LD had the highest AUCs among all LD parameters in these standing conditions, with the highest sensitivity during the N-EO condition. Meanwhile, the velocity-ML of CoP parameters was similar between two age groups during the W-EO and velocity-AP of CoP had a higher AUC than velocity-ML of CoP to differentiate the two age groups during wide-based standing conditions. In wide-based standing, postural control was mainly achieved through ankle strategies, which could be better reflected by AP displacement of the CoP [39]. The above findings implied a lower sensitivity of the CoP parameters than the LD parameters to capture the age-related change in ML control without increased demand for postural control to amplify the age-related effects [4, 40].

### Study limitation

This study has several limitations that should be addressed. First, we analyzed only five LD parameters, including time-domain distance and area measures among a large number of measures [10]. These parameters are estimates of the size of the stabilogram and the control effectiveness of control system, and have been commonly used to detect age-related changes. However, there are quite a few more parameters, such as frequency domain or time domain hybrid measures, or nonlinear metrics, which can provide different insights into the strategies used in different stance conditions. Second, we did not adjust for some demographic characteristics, such as body height or body weight, which may affect postural steadiness. The reason for not including these two factors is that they are significantly correlated with age. With the current number of participants, it is difficult to perform a subgroup analysis. Third, the demographics of the current participants, who were mainly healthy adults, likely limited the generalizability of the findings to subjects with impaired balance. A previous study included young, elderly, and poststroke individuals and showed different postural control strategies in the three groups in the frontal and sagittal planes according to CoP-CoM analysis [41]. Therefore, additional studies are warranted to help determine the feasibility of this system with disease-specific populations.

## Conclusion

The design of our system that used a lumbar tracker to record the LD positions was similar to the design of a swaymeter to record trunk sway [23, 42] but was advantageous to allow digital recordings without restricting body motion. The LD parameters obtained from a VIVE tracker-based system, as well as CoP parameters, could differentiate age-related changes in postural steadiness in four stance conditions. In addition, LD parameters discriminated the two age groups better than CoP parameters with velocity-ML in all standing conditions. The study results supported the use of this system to explore age-related changes in postural steadiness and can provide complimentary information regarding the control strategies used by the elderly. Additional studies should be conducted to determine the link between the CoP and LD parameters and fall events and help clarify the predictive validity of the above measures for fall risk classification.

## Data Availability

The datasets used and/or analysed during the current study The datasets generated and/or analyzed during the current study are not publicly available due to the restriction under the institutional ethical committee ‘s policy, but may be available from the corresponding author on reasonable request and with permission of the ethical committee.

## Abbreviations

AP: Anterior–posterior
AREA-CE: 95% Confidence ellipse area
AUC: Area under the curve
BMI: Body mass index
CoG: Center of gravity
CoM: Center of mass
CoP: Center of pressure
EC: Eyes closed
EO: Eyes open
ICC: Intraclass correlation coefficient
LD: Body displacement at lumbar level
ML: Medial–lateral
N-EC: Narrow-stance with eyes closed
N-EO: Narrow-stance with eyes open
RMS: Root mean square
ROC: Receiver operating characteristic
VR: Virtual reality
W-EC: Wide-stance with eyes closed
W-EO: Wide-stance with eyes open
